# HLA Expression at the Maternal-Fetal Interface

**DOI:** 10.1155/1998/65065

**Published:** 1998

**Authors:** Heinz Hutter, Astrid Hammer, Gottfried Dohr, Joan S. Hunt

**Affiliations:** 1 Department of Histology and Embryology University of Graz Graz A-8010 Austria; 2 Department of Anatomy and Cell Biology University of Kansas Medical Center Kansas City Kansas 66160-7400 USA; 3 Department of Histology and Embryology Karl-Fransens University of Graz Harrachgasse 21/7 Graz A-8010 Austria

**Keywords:** MHC, HLA class l, placenta, trophoblast, immunology

## Abstract

Pregancy in the human presents an “immunological paradox,” because of the unexpected willingness of mothers to accept genetically disparate tissues. The fact that the fetus can develop unharmed for nine months shows that protective mechanisms must exist to permit its survival. The conditions that permit the genetically dissimilar human fetus to evade rejection by its mother's immune system have been the subject of intense interest for several decades. As the placental cells, which are in contact with maternal blood or tissue, are devoid of HLA class II antigens, interest has focused on the expression of HLA class molecules. Recent developments in the constitutive, transcriptional, and translational expression of HLA class I molecules on anatomically and morphologically different subpopulations of trophoblast cells will form the basis of this short review.


*Developmental Immunology*, 1998, Vol. 6, pp. 197–204
Reprints available directly from the publisher
Photocopying permitted by license only

© 1998 OPA (Overseas Publishers Association)
N.V. Published by license under the
Harwood Academic Publishers imprint,
part of the Gordon and Breach Publishing Group
Printed in Malaysia

*Minireview*

### HLA Expression at the Maternal-Fetal Interface

HEINZ HUTTER[*,a,†], ASTRID HAMMER[a], GOTTFRIED DOHR[a] and JOAN S. HUNT[b]

[a]*Department of Histology and Embryology, University of Graz, A-8010 Graz, Austria*; [b]*Department of Anatomy and Cell Biology, University of Kansas Medical Center, Kansas City, Kansas 66160-7400*

(*Received 21 October 1996*; *Accepted 9 May 1997*)

Pregancy in the human presents an "immunological paradox," because of the unexpected willingness of mothers to accept genetically disparate tissues. The fact that the fetus can develop unharmed for nine months shows that protective mechanisms must exist to permit its survival. The conditions that permit the genetically dissimilar human fetus to evade rejection by its mother's immune system have been the subject of intense interest for several decades. As the placental cells, which are in contact with maternal blood or tissue, are devoid of HLA class II antigens, interest has focused on the expression of HLA class I molecules. Recent developments in the constitutive, transcriptional, and translational expression of HLA class I molecules on anatomically and morphologically different subpopulations of trophoblast cells will form the basis of this short review.

*Keywords:* MHC, HLA class I, placenta, trophoblast, immunology

### INTRODUCTION

The human major histocompatibility complex region (MHC) spans about 4000 kb in the distal region of the short arm of chromosome 6, containing class I, II, and III subregions. The class I subregion is larger than the two others and encodes cell-surface glycoproteins of m.w. 40 to 45 kD that associate noncovalently with the 12-kD $\beta_2$-microglobulin ($\beta_2$-m) light chain. These include the three polymorphic molecules HLA-A, -B, and -C, which are ubiquitously expressed and are able to give rise to immune reactions by presenting peptides from intracellularly degraded proteins on the cell surface (Le Bouteiller, 1994). They allow discrimination of "self" from "nonself" and serve as restriction elements for virus-specific and allo-specific T lymphocytes. In addition, they affect the activities of natural killer (NK) cells.

Three additional class I genes commonly referred as nonclassical or class Ib genes, all highly homologous to the other class I genes and all of which associate with $\beta_2$-m, complete this gene family

*Corresponding author.
†Present address: Department of Histology and Embryology, Karl-Fransens University of Graz, Harrachgasse 21/7, A-8010 Graz, Austria.

(Geraghty et al., 1987, 1990; Koller et al., 1988; Le Bouteiller, 1994). In humans, each of the class Ib genes appears to exhibit a distinct pattern of expression in developing and adult tissues. HLA-E transcripts are distributed widely in adult tissues and have also been found in the placenta and fetal liver (Wei and Orr, 1990; Houlihan et al., 1992). HLA-E can be regarded as ubiquitous, like the classical HLA class I molecules. The HLA-E gene is thought to represent an ancient gene as there are data suggesting that the polymorphism at the HLA-E locus may have existed before the appearance of most HLA-A, -B, and -C polymorphism (Geraghty et al., 1992). Furthermore, this locus is conserved between macaques and humans (Boyson et al., 1995). Such a maintenance through evolution is likely to reflect a significant and important function; however, this function is still unknown. The group headed by P. Le Bouteiller (Boucrout et al., 1993) suggests that HLA-E may have functional importance as a housekeeping gene, as it is expressed as the only HLA class I molecule in the trophoblast-derived cell line JAR.

In the adult, the presence of HLA-F has been shown in skin, resting T cells, and B cells (Geraghthy et al., 1990), whereas in development, its expression was reported in fetal liver (Houlihan et al., 1992) and in low levels in placenta and extraplacental tissues (Wei and Orr, 1990).

HLA-G is the most interesting nonclassical HLA class I molecule, because of its exceptional high expression at the maternal-fetal interface, suggesting that HLA-G plays a critical role in human pregnancy. In contrast to classical HLA class I genes, the primary HLA-G transcript is alternately spliced, giving rise to at least five distinct mRNAs, named HLA-G1 to HLA-G5 (Ishitani and Geraghty, 1992; Fujii et al., 1994; Kirszenbaum et al., 1994; Moreau et al., 1995). These mRNAs transcribe membrane-bound and soluble forms of HLA-G antigens (for a recent review, see Carosella et al., 1996). The relative levels of the alternate mRNAs are affected by the cell type and the developmental stage of the expressing tissue (Ishitani and Geraghty, 1992). However, because of the lack of antibodies recognizing the different isoforms, it is not clear to what extent these alternately spliced mRNAs are translated to protein. Most of the reports of HLA-G expression refer to HLA-G1, which possesses a full-length mRNA, giving rise to a protein containing all domains. The HLA-G1 protein product has 86% sequence identity with the class I consensus sequence (Parham et al., 1988). HLA-G was shown to be synthesized as five 37- to 39-kD isoforms, one of which is secreted (Kovats et al., 1990).

Messenger RNA for HLA-G has been detected in several embryonic and adult tissues, including adult and fetal thymus (Wei and Orr, 1990), fetal liver and eye (Shukla et al., 1990; Houlihan et al., 1992), adult spleen (Onno et al., 1994), keratinocytes (Ulbrecht et al., 1994), but the protein was reported to be restricted to several populations of trophoectodermal-derived trophoblast and choriocarcinoma cells (Kovats et al., 1990; Chumbley et al., 1994a). However, HLA-G protein was also found recently on embryoblast-derived amnion epithelial cells and in the amnionic fluid (Hammer et al., 1996) and in human mononuclear phagocytes (Yang et al., 1996).

### THE MATERNAL-FETAL INTERFACE

The maternal-fetal interface extends over almost the entire surface of the fetal membranes and the placenta. During the course of pregnancy, the fetus itself does not come into direct contact with maternal tissue; it is the trophoblast, which constitutes the effective intrauterine tissue allograft and forms the interface between the maternal and fetal compartments (Benirschke and Kaufmann, 1995). Within the membranes, extravillous cytotrophoblast cells of the chorion laeve are in close contact with maternal cells of the decidua capsularis. As pregnancy goes on, they are additionally in contact with maternal cells of the decidua parientalis. Within the placenta, the syncytiotrophoblast, which overlays a layer of villous cytotrophoblast cells, forms the outer surface of the chorionic villi, directly facing the maternal blood that perfuses the intervillous space.

The basal plate is an intimate contact zone of maternal and fetal tissues. The early precursor of the basal plate is the trophoblastic shell. During implantation, it consists merely of syncytiotrophoblast. With the formation of chorionic villi, increasing amounts of cytotrophoblast cells come into contact with the maternal decidua basalis. These extravillous cytotrophoblast cells proliferate within the cell columns of anchoring villi, and subsequently leave the proliferation zones, and deeply invade maternal tissue and even maternal blood vessels. By considering morphology and anatomical location, different subsets of invasive extravillous cytotropblast cells may be distinguished: Interstitial trophoblast cells are interposed between maternal cells of the decidua basalis outside of the uteroplacental vessels. Intramural trophoblast cells are incorporated into the arterial walls, and intraluminal trophoblast cells are located directly within the lumen of the uteroplacental arteries. All of these extravillous cytotrophoblast cells are in intimate contact with a variety of maternal tissues, namely, uterine glandular cells, stromal cells, endothelial cells, and maternal leukocytes, and with maternal blood. The maternal leukocytes present in human decidua during the early weeks of pregnancy are composed of $CD56^+$ NK-like cells (~80%), $CD3^+$ T cells (~10%), and $CD14^+$ macrophages (~10%) (King et al., 1989; Bulmer et al. 1991). In the basal plate, the contact between fetal and maternal cells is most intimate during the invasive phase at the onset of pregnancy; later the two cell populations are separated mostly by fibrinoid.

### HLA CLASS I EXPRESSION IN THE PLACENTA

Anatomically and morphologically distinct subpopulations of trophoblast cells contain different levels of HLA class I mRNA and express the protein antigen differently from one another. These antigens are either totally inhibited or are carefully selected from among the many members of the class I multigene family. Furthermore, within a given trophoblast cell population, the expression is dependent on the stage of gestation; generally, first-trimester trophoblast cells transcribe more HLA class I mRNA (Wei and Orr, 1990) and contain more HLA class I protein (Kovats et al., 1990) than term trophoblast cells. The differentiated expression of HLA class I molecules in trophoblast cells requires several controlling mechanisms both on the transcriptional as well as on the translational level; for recent reviews referring to this topic, see Le Bouteiller et al. (1996) and Hunt and Hutter (1996). On the basis of the more recent data, the constitutive, transcriptional, and translational expression of classical and nonclassical HLA class I molecules in the different trophoblast-cell subpopulations that constitute the materno-fetal interface during human pregnancy is as follows.

Most syncytiotrophoblast lacks HLA class I mRNA and, if these molecules are transcribed at all, the transcription is very low. Low amounts of HLA class I mRNA were shown by *in situ* hybridization on first-trimester syncytiotrophoblast (Hunt et al., 1988), which were identified later to be HLA-G (Yelavarthi et al., 1991). In syncytiotrophoblast of term placenta, the presence of HLA-A and -B/-C mRNA has been described (Guillaudeux et al., 1995). However, the experiments for the detection of the mRNA for HLA-A, -B/-C were carried out on an *in vitro* differentiated syncytiotrophoblast derived from villous cytotrophoblast cells and, as the authors state, the possibility that the HLA transcripts derived from cell contamination cannot be excluded. It is not clear in general whether HLA class I mRNA detected in syncytiotrophoblast is truly synthesized or the message is possibly donated by merging cytotrophoblast cells (Hunt et al., 1990). The *in vivo* syncytiotrophoblast is reported to lack the expression of HLA class I protein; and in fact, we and others have not been able to find any HLA class I antigen with the antibodies available so far. However, preliminary results from Ishitani et al. (1996) carried out with a new antibody show a staining for soluble HLA-G. As the transcription for HLA class I molecules in syncytiotrophoblast is very low, the source of this antigen is not clear and further experiments have to be done to clarify the situation.

Villous cytotrophoblast cells are known to transcribe class I molecules, but the transcription seems to be not homogenous. *In situ* hybridization with first-trimester specimens showed that in many villi, the villous cytotrophoblast cells did not contain HLA-G mRNA (Yelavarthi et al., 1991). The nonclassical HLA class I molecules HLA-E and -F were not found in villous cytotrophoblast cells of first-trimester specimens by *in situ* hybridization (Yelavarthi et al., 1991; Chumbley et al., 1993); the only HLA-E-positive cells were identified morphologically as lymphocytes. However, two transcripts of HLA-E and three isoforms of HLA-G were found by Northern blotting and Reverse Transcription-Polymerase Chain Reaction (RT-PCR), respectively, in purified term villous cytotrophoblast (Guillaudeux et al., 1995).

Among the classical HLA class I genes, mRNA specific for HLA-C in first-trimester villous cytotrophoblast cells and HLA-A and possibly also -B in term villous cytotrophoblast cells could be demonstrated by RT-PCR and Northern blotting, respectively (King et al., 1996a). The expression of HLA-B, however, is doubtful because the probe used by Guillaudeux et al. (1995) is known to cross hybridize with HLA-C even under stringent hybridization conditions. Therefore, it is possible that, in fact, HLA-C was detected. Immunohistochemical staining with different antibodies against HLA class I heavy chains or heavy chains associated with $\beta_2$-m failed to react with villous cytotrophoblast cells. Recently, Rodriguez et al. (1996) reported that classical HLA class I heavy chains remain unfolded in the endoplasmatic reticulum of villous cytotrophoblast cells through the association with calnexin due to a posttranslational defect involving the peptide transporter system. After treatment with IFN-$\gamma$, part of these class I heavy chains leave the endoplasmatic reticulum and become detectable at the cell surface. Reports on immunoprecipitation experiments using purified cytotrophoblast cells show the presence of a 39- and a 45-kD band (Kovats et al., 1990; Le Bouteiller et al., 1996). However, the detected proteins seem to be derived either from contaminations with a cell-island trophoblast (which is prominent in preparations of first- and second-trimester villous cytotrophoblast cells) or is due to cell-culture conditions.

Extravillous cytotrophoblast (eCT) cells located in the basal plate, cell islands, chorionic plate, and fetal membranes share two common features. Those cells resting on the basal lamina facing the chorionic mesoderm (chorionic plate, membranes) or the villous stroma (cell columns, cell islands) seem to be the proliferating stem cells, whereas the nonproliferating cells represent more highly differentiated, invasive daughter cells of the former (Benirschke and Kaufmann, 1995). The differences between proliferating and invasive extravillous cytotrophoblast cells are not only illustrated by a different distribution of EGFR and c-erbB2 (Mühlhauser et al., 1993; Jokhi et al., 1994), but also by a different distribution of HLA class I molecules.

Invasive eCT cells in the cell islands, placental septa, and the basal plate, like interstitial, intramural, and intraluminal cytotrophoblast cells as well as trophoblastic giant cells, have been shown to express HLA-G and additionally, as has been previously stated, the classical HLA-C molecule (Chumbley et al., 1994a; King et al., 1996a; Hutter et al., 1996). The nature of the origin of the trophoblastic giant cells is controversial. Nevertheless, these cells express HLA-G and -C antigens. However, Hunt et al. (1991) reported low amounts of HLA class I mRNA in these cells compared with other interstitial trophoblast.

In cell columns and cell islands, the expression of HLA class I molecules is not homogenous. We could show (Hutter et al., 1996) that cells in the proliferative zone, proximal to the villous core, do not contain the HLA-G antigen, but become positive for these molecules in the distal part of the cell columns as the cytotrophoblast cells comes into contact with the uterine wall. Our results confirm findings obtained by McMaster et al. (1995), who describe the upregulation of HLA-G antigen as an integral part of cytotrophoblast differentiation along the invasive pathway. In contrast to HLA-G, the HLA-C antigen could be detected in the proliferative as well as in the invasive zone (Hutter et al., 1996). However, Shorter et al. (1993) reported a complete lack of antigen staining in the proliferative zone with W6/32. These results cannot be confirmed by our own observations. W6/32 and a serum against $\beta_2$-m did show a less pronounced signal in the proliferative than in the invasive zone; nevertheless, there was a clearly detectable signal. Furthermore, an antibody against

HLA-B and -C did not show differences in the staining intensity between the proliferative and invasive zones (Hutter et al., 1996). Our findings are in agreement with results from Chumbly et al. (1994a), who detected a cell subpopulation of first-trimester extravillous cytotrophoblast cells reacting with W6/32 in fluorescence-activated cell sorter (FACS) analysis, but not with a polyclonal antibody against HLA-G.

In the fetal membranes, the mRNA for HLA-E and -G but not for HLA-F can be detected by RNAse protection analysis (Wei and Orr, 1990). Unfortunately, as the authors worked with the whole tissue containing trophoblast cells, mesenchymal cells, decidua cells, and amnionic epithelium cells, the source of the message cannot be established. *In situ* hybridization, carried out by Yelavarthi et al. (1991), detected the mRNA for HLA-G but not for HLA-E in extravillous cytotrophoblast cells of term membranes. However, Houlihan et al. (1995) recently reported the detection of HLA-E mRNA and the respective protein in amnionic epithelial cells. Immunohistochemically HLA-G was the only HLA class I molecule that could be detected in extravillous chorionic cytotrophoblast cells (Shorter et al., 1993; Hutter et al., 1996). In contrast to these findings, amnionic epithelial cells, derived from the inner cell mass of the blastocyste, express all of the classical HLA class I antigens along with HLA-G and HLA-E (Houlihan et al., 1995; Hammer et al., 1997).

### POSSIBLE FUNCTIONS OF HLA CLASS I MOLECULES

Although several functions have been proposed both for HLA-G and HLA-C, their role on trophoblast cells remains a matter of speculation. Two different types of immunological functions can be envisaged for HLA-C and HLA-G. The first is a conventionally active role in alerting T cells to infection and other trauma that befall trophoblast cells. Like the classical HLA class I molecules, HLA-G expressed by transfected LCL.221 cells consists of heavy and light chains complexed with nonameric endogenous peptides in a 1:1:1 ratio (Lee et al., 1995). Furthermore, as was shown by a transient cell-cell adhesion assay, HLA-G is able to bind to a $\alpha/\alpha$ homodimer of the CD8 receptor that is expressed on T lymphocytes and some NK cells (Sanders et al., 1991). This enables HLA-G to act like a classical HLA class I molecule in presenting peptides to allo-specific T cells. The $\gamma/\delta$ T-cell receptor (TcR) may be the ligand for HLA-G (Kovats et al., 1991; Houlihan et al., 1992).

The second immunological function is an inhibitory role in preventing the activation of allo-reactive T and NK cells; the latter preferentially kill target cells with absent or low levels of HLA class I molecules. CD56$^+$ NK cells infiltrate the decidua at the onset of pregnancy in large numbers. Ferry et al. (1991) have shown that extravillous cytotrophoblasts are resistant to both decidual and peripheral blood NK cells even after activation of these large granular lymphocytes (LGL) with interleukin-2. Furthermore, Chumbley et al. (1994b) showed that HLA-G$^+$ LCL.221 transfectants are partially resistant to lysis by decidual CD56$^+$ NK, although this protection was not as marked as that conferred by HLA-A2. This finding suggests that HLA-G as well as HLA-C takes part in the resistance to NK cell attack. An interesting assumption was made by Loke and King (1991), who suggested that the CD56$^+$ NK cells play a role in the control of implantation by limiting trophoblast migration into maternal decidua and spiral arteries with trophoblast HLA-G as possible regulatory molecules.

The most interesting point—when comparing pregnancy with a successful tissue allograft—is how trophoblast cells, expressing HLA-G and HLA-C, escape recognition and subsequent destruction by allo-reactive and HLA-C-specific T cells. HLA-G is more polymorphic than initially assumed (Van der Ven and Ober, 1994). Nevertheless, extravillous cytotrophoblast cells from first-trimester chorionic tissue were not able to stimulate the proliferation of purified decidual leukocytes nor of peripheral blood lymphocytes (King et al., 1996b). Possibly the soluble forms of HLA-G might play an important role in suppressing maternal cytotoxic T cells. Soluble HLA-G molecules derived from transfected LCL.221 cells show the same motif of bound peptides as the membrane bound forms on the same cells (Lee et al., 1995). Therefore, soluble HLA-G could bind either directly to the TcR or alternatively to CD8 on the T cell and thereby inhibit the interaction between T cell and class I antigens on target cells. Furthermore, new observations from Puppo et al. (1996) report that phythaemagglutinin-activated $CD8^+$ T cells undergo apoptosis when incubated with purified soluble HLA class I molecules from human plasma.

Beside the immunological function of the HLA class I molecules, an involvement in the key functions of ligand-activated receptors has been suggested. In mice, structural associations of HLA class I molecules with receptors for epidermal growth factor and insulin (Schreiber et al., 1984; Fehlmann et al., 1985; Due et al., 1986), as well as for luteinizing hormone receptors and beta-adrenergic receptors (Solano et al., 1988), have been reported. A 25-residue peptide binding to murine MHC-I on the cell surface, not in the groove but apparently to the $\alpha 1$ helix, inhibits internalization of certain receptors like these for insulin and glucose, thereby increasing the steady-state number of active receptors on the cell surface (Stagsted et al., 1990; Olsson et al., 1994). Furthermore Solano et al. (1988) have suggested that MHC class I interaction with peptide hormone receptors is necessary to produce the microaggregation of the hormone receptor thought to be essential for peptide hormone action.

### CONCLUSION

HLA class I molecules on the maternal-fetal interface are differentially expressed by trophoblast subpopulations. According to the trophoblast subpopulation and stage of gestation, a minimal transcription up to rather high amounts of translated protein can be observed. Extravillous cytotrophoblast cells are the only trophoblast cells expressing membrane-bound HLA class I molecules associated with $\beta_2$-m *in vivo*. Membrane-bound HLA-G can be detected in extravillous trophoblast cells with invasive properties. In contrast to HLA-G, HLA-C is restricted to extravillous trophoblast cells of the basal plate and cell islands, where it can be found in dividing as well as in invasive cells. HLA-C cannot be detected in the chorionic extravillous trophoblast cells of the fetal membranes. As soluble HLA-G was found even in syncytiotrophoblast, further investigations will be necessary to determine the different functions of soluble and membrane-bound HLA class I molecules on trophoblast cells.

### *Acknowledgements*

This work was supported by a grant from the FWF (P09922 Med.), a grant from the European Community (PL941593), both awarded to G. Dohr and the Franz Lanyar Stiftung. Studies in the Hunt laboratory are supported by N.I.H. grant HD 25691. The authors thank A. Blaschitz for excellent technical assistance.

### *References*

Benirschke K., and Kaufmann P. (1995). Pathology of the Human Placenta, 3rd ed. (New York: Springer).

Boucraut J., Guillaudeux T., Alizadeh M., Boretto J., Chimini G., Malecaze F., Semana G., Fauchet R., Pontarotti P., and Le Bouteiller P. (1993). HLA-E is the only class I gene that excapes CpG methylation and is transcriptionally active in the trophoblast-derived human cell line JAR. Immunogenetics **38**: 117-130.

Boyson J.E., McAdam S.N., Gallimore A., Golos T.G., Liu X., Gotch F.M, Hughes A.L., and Watkins D.I. (1995). The MHC-E locus in macaques is polymorphic and is conserved between macaques and humans. Immunogenetics **41**: 59-68.

Bulmer J.N., Morrison L., Longfellow M., Ritson A., and Pace D. (1991). Granulated lymphocytes in human endometrium: Histochemical and immunohistochemical studies. Human Reprod. **6**: 791-798.

Carosella E., Dausset J., and Kirszenbaum M. (1996). HLA-G revisited. Immunol. Today **17**: 407-409.

Chumbley G., King A., Holmes N., and Loke Y.W. (1993). In situ hybridization and Northern blot demonstration of HLA-G mRNA in human trophoblast populations by locus-specific oligonucleotide. Human Immunol. **37**: 17-22.

Chumbley G., King A., Gardner L., Howlett S., Holmes N., and Loke Y.W. (1994a). Generation of an antibody to HLA-G in transgenic mice and demonstration of the tissue reactivity of this antibody. J. Reprod. Immunol. **27**: 173-186.

Chumbley G., King A., Robertson K., Holmes N., and Loke Y.W. (1994b). Resistance of HLA-G and HLA-A2 transfectants to lysis by decidual NK cells. Cell. Immunol. **155**: 312-322.

Due C., Simonsen M., and Olsson L. (1986). The major histocompatibility complex class I heavy chain as a structural subunit of the human cell membrane insulin receptor: Implications for the range of biological functions of histocompatibility antigens. Proc. Natl. Acad. Sci. USA **83**: 6007-6011.

Fehlmann M., Peyron J.F., Samson M., Van-Obberghen E., Brandenburg D., and Brossette N. (1985). Molecular association between major histocompatibility complex class I antigens and insulin receptors in mouse liver membranes. Proc. Natl. Acad. Sci. USA **82**: 8634-8637.

Ferry B.L., Sargent I.L., Starkey P.M., and Redman C.W.G. (1991). Cytotoxic activity against trophoblast and choriocarcinoma cells of large granular lymphocytes from human early pregnancy decidua. Cell. Immunol. **132**: 140-149.

Fujii T., Ishitani A., and Geraghty D.E. (1994). A soluble form of the HLA-G antigen is encoded by a messenger ribonucleic acid containing intron 4. J. Immunol. **153**: 5516-5524.

Geraghty D.E., Koller B.H., and Orr H.T. (1987). A human major histocompatibility complex class I gene that encodes a protein with a shortened cytoplasmic segment. Proc. Natl. Acad. Sci. USA **84**: 9145-9149.

Geraghty D.E., Stockschleader M., Ishitani A., and Hansen J.A. (1992). Polymorphism at the HLA-E locus predates most HLA-A and -B polymorphism. Human Immunol. **33**: 174-184.

Geragthy D.E., Wei X., Orr H.T., and Koller B.H. (1990). HLA-F: An expressed HLA gene composed of a class I coding sequence linked to a novel repetitive element. J. Exp. Med. **171**: 1-18.

Guillaudeux T., Rodriguez A.M., Girr M., Mallet V., Ellis S.A., Sargent I.L., Fauchet R., Alsat E., and Le Bouteiller P. (1995). Methylation status and transcriptional expression of the MHC class I loci in human trophoblast cells from term placenta. J. Immunol. **154**: 3283-3299.

Hammer A., Hutter H., Blaschitz A., Mahnert W., Hartmann M., Uchanska-Ziegler B., Ziegler A., and Dohr G. (1997). Amnion epithelial cells, in contrast to trophoblast cells, express all classical HLA class I molecules together with HLA-G. Amer. J. Reprod. Immunol. **137**:161–171.

Houlihan J.M., Biro P.A., Fergar-Payner A., Simpson K.L., and Holmes C.H. (1992). Evidence for the expression of non-HLA-A, -B, -C class I genes in the human fetal liver. J. Immunol. **149**: 668-675.

Houlihan J.M., Biro P.A., Harper H.M., Jenkinson H.J., and Holmes C.H. (1995). The human amnion is a site of MHC class Ib expression: Evidence for the expression of HLA-E and HLA-G. J. Immunol. **154**: 5665-5674.

Hunt J.S., Fishback J.L., Andrews G.K., and Wood G.W. (1988). Expression of class I HLA genes by trophoblast cells: Analysis by in situ hybridization. J. Immunol. **140**: 1293-1299.

Hunt J.S., Fishback J.L., Chumbley G., and Loke Y.W. (1990). Identification of class I MHC mRNA in human first trimester trophoblast cells by in situ hybridization. J. Immunol. **144**: 4420-4425.

Hunt J.S., Hsi B.L, King C.R., and Fishback J.L. (1991). Detection of class I MHC mRNA in subpopulations of first trimester cytotrophoblast cells by in situ hybridization. J. Reprod. Immunol. **19**: 315-332.

Hunt J.S., and Hutter H. (1996). Current theories on the protection of the featal semialograft. In HLA and the Maternal-Fetal Realtionship, Hunt J.S., Ed. (Austin, Tx: R.G. Landes), pp. 27-50.

Hutter H., Hammer A., Blaschitz A., Hartmann M., Ebbesen P., Dohr G., Ziegler A., and Uchanska-Ziegler B. (1996). Expression of HLA class I molecules in human first trimester and term placenta trophoblast. Cell Tissue Res. **286:** 439–447.

Ishitani A., Dorofeeva N., Lee N., Kapasi K., Hunt J.S., and Geraghty D.E. (1996). A set of antibodies which specifically bind alternative HLA-G proteins distinguish HLA-G expression in vivo. Human Immunol. **47**: 144.

Ishitani A., and Geraghty D.E. (1992). Alternative splicing of HLA-G transcripts yields proteins with primary structures resembling both class I and class II antigen. Proc. Natl. Acad. Sci. USA **89**: 3947-3951.

Jokhi P.P., King A., and Loke Y.W. (1994). Reciprocal expression of epidermal growth factor receptor (EGF-R) and c-erbB2 by non-invasive and invasive human trophoblast populations. Cytokine **6**: 433-442.

King A., Birkby C., and Loke Y.W. (1989). Early human decidual cells exhibit NK activity against K 562 cell line but not against first trimester trophoblast. Cell. Immunol. **118**: 337-344.

King A., Boocock C., Sharkey A.M., Gardner L., Beretta A., Siccardi A.G., and Loke Y.W. (1996a). Evidence for the expression of HLA-C class I mRNA and protein by human first trimester trophoblast. J. Immunol. **156**: 2068-2076.

King A., Gardner L., and Loke Y.W. (1996b). Human decidual leukocytes do not proliferate in response to either extravillous trophoblast or allogeneic peripheral blood lymphocytes. J. Reprod. Immunol. **30**: 67-74.

Kirszenbaum M., Moreau P., Gluckman E., Dausset J., and Carosella E. (1994). New alternatively spliced form of HLA-G mRNA in human trophoblasts and evidence for the presence of HLA-G transcripts in adult lymphocytes. Proc. Natl. Acad. Sci. USA **91**: 4209-4213.

Koller B.H., Geraghty D.E., Shimizu Y., DeMars R., and Orr H.T. (1988). A novel HLA class I gene expressed in resting T lymphocytes. J. Immunol. **141**: 897-904.

Kovats S., Main E.K., Librach C., Fisch P., Main E.K., Sondel P.M., Fisher S.J., and DeMars R. (1991). Expression and possible functions of the HLA-G a chain in human cytotrophoblasts. In Cellular and Molecular Biology of the Materno-Fetal Relationship, Vol. 212, Chaouat J., and Mowbray J., Eds. (New York: John Libbey Eurotext), pp. 21-29.

Kovats S., Main E.K., Librach C., Subblebine M., Fisher S.J., and DeMars R. (1990). A class I antigen, HLA-G, expressed in human trophoblasts. Science **248**: 220-223.

Le Bouteiller P. (1994). HLA class I chromosomal region, genes, and products: Facts and questions. Crit. Rev. Immunol. **14**: 89-129.

Le Bouteiller P., Rodriguez A.M., Mallet V., Girr M., Guillaudeux T., and Lenfant F. (1996). Placental expression of HLA class I genes. Amer. J. Reprod. Immunol. **35**: 216-225.

Lee N., Malacko A.R., Ishitani A., Chen M.C., Bajorath J., Marquardt H., and Geraghty D.E. (1995). The membrane-bound and soluble forms of HLA-G bind identical sets of endogenous peptides but differ with respect to TAP association. Immunity **3**: 591-600.

Loke Y.W., and King A. (1991). Recent developments in the human maternal-fetal immune interaction. Curr. Opin. Immunol. **3**: 762-766.

McMaster M.T., Librach C.L., Zhou Y., Lim K.H., Janatpour M.J., DeMars R., Kovats S., Damsky C., and Fisher S.J. (1995). Human placental HLA-G expression is restricted to differentiated cytotrophoblasts. J. Immunol. **154**: 3771-3778.

Moreau P., Carosella E., Teyssier M., Prost S., Gluckman E., Dausset J., and Kirszenbaum M. (1995). Soluble HLA-G molecule. An alternatively spliced HLA-G mRNA form candidate to encode it in peripheral blood mononuclear cells and human trophoblasts. Human Immunol. **43**: 231-236.

Mühlhauser J., Crescimanno C., Kaufmann P., Höfler H., Zaccheo D., and Castellucci M. (1993). Differentiation and proliferation patterns in human trophoblast revealed by c-erbB-2 oncogene product and EGF-R. J. Histochem. Cytochem. **41**: 165-173.

Olsson L., Goldstein A., and Stagsted J. (1994). Regulation of receptor internalization by the major histocompatibility complex class I molecule. Proc. Natl. Acad. Sci. USA **91**: 9086-9090.

Onno M., Guillaudeux T., Amiot L., Renard I., Drenau B., Hirel B., Girr M., Semana G., Le Bouteiller P., and Fauchet R. (1994). The HLA-G gene is expressed at a low mRNA level in different human cells and tissues. Human Immunol. **41**: 79-86.

Parham P., Lomen C.E., Lawlor D.A., Ways J.P., Holmes N., Coppin H.L., Salter R.D., Wan A.M., and Ennis P.D. (1988). Nature of polymorphism in HLA-A, -B and -C molecules. Proc. Natl. Acad. Sci. USA **85**: 4005-4009.

Puppo F., Cantini P., Chio M., Brenci S., Bosco O., Lanza L., Scudelette M., and Indiveri F. (1996). Soluble HLA class I molecules induce apoptosis in human PHA-Activated $CD8^+$ T lymphocytes. Human Immunol. **47**: 150.

Rodriguez A.M., Mallet V., Lenfant F., and Le Bouteiller P. (1996). Interferon-g rescues HLA class Ia cell surface expression in term villous trophoblast cells by inducing synthesis of TAP proteins. Human Immunol. **47**: 144.

Sanders S.K., Giblin P.A., and Kavathas P. (1991). Cell-cell adhesion mediated by CD8 and human histocompatibility leukocyte antigen G, a nonclassical major histocompatibility complex class I molecule on cytotrophoblasts. J. Exp. Med. **174**: 737-740.

Schreiber A.B., Schlessinger J., and Edidin M. (1984). Interaction between major histocompatibility complex antigens and epidermal growth factor receptors on human cells. J. Cell. Biol.**98**: 725-731.

Shorter S.C., Starkey P.M., Ferry B.L., Clover L.M., Sargent I.L., and Redman C.W.G. (1993). Antigenic heterogeneity of human cytotrophoblast and evidence for the transient expression of MHC class I antigens distinct from HLA-G. Placenta **14**: 571-582.

Shukla H., Swaroop A., Srivastava R., and Weissman S.M. (1990). The mRNA of a human class I gene HLA-G/HLA 6.0 exhibits a restricted pattern of expression. Nucleic Acids Res. **18**: 2189.

Solano A.R., Cremaschi G., Sanchez M.L., Borda E., Sterin-Borda L., and Podesta E.J. (1988). Molecular and biological interaction between major histocompatibility complex class I antigens and luteinizing hormone receptors or beta-adrenergic receptors triggers cellular response in mice. Proc. Natl. Acad. Sci. USA **85**: 5087-5091.

Stagsted J., Reaven G.M., Hansen T., Goldstein A., and Olsson L. (1990). Regulation of insulin receptor functions by a peptide derived from a major histocompatibility complex class I antigen. Cell **62**: 297-307.

Ulbrecht M., Rehberger B., Strobel I., Messer G., Kind P., Degitz K., Biber T., and Weiss E.H. (1994). HLA-G: Expression in human keratinocytes in vitro and in human skin in vivo. Eur. J. Immunol. **24**: 176-180.

Van der Ven K., and Ober C. (1994). HLA-G polymorphisms in African Americans. J. Immunol. **153**: 5628-5633.

Wei X., and Orr H.T. (1990). Differential expression of HLA-E, HLA-F, and HLA-G transcripts in human tissue. Human Immunol. **29**: 131-142.

Yang Y., Chu W., Geraghty D.E., and Hunt J.S. (1996). Expression pf HLA-G in human mononuclear phagocytes and selective induction by IFN-gamma. J. Immunol. **156**: 4424-4231.

Yelavarthi K.K., Fishback J.L., and Hunt J.S. (1991). Analysis of HLA-G mRNA in human placental and extraplacental membrane cells by in situ hybridization. J. Immunol. **146**: 2847-2854.

